# Targeting glioblastoma with HDAC inhibitors: insights into hydroxamic acid-based therapeutic strategies

**DOI:** 10.1186/s40478-025-02194-7

**Published:** 2025-12-02

**Authors:** Padmini Pai, Ipshita Das, Yashaswini Reddy, Babu Santhi Venkidesh, Poonam Bhandari, Manjunath Madalageri, Veeresh Sadashivanavar, Karkala Sreedhara Ranganath Pai, Pallavi Rao, Srinivas Oruganti, Manasa Gangadhar Shetty, Kapaettu Satyamoorthy, Babitha Kampa Sundara

**Affiliations:** 1 Department of Biophysics, Manipal School of Life Sciences, Manipal Academy of Higher Education, Manipal, Karnataka 576104 India; 2 Department of Radiation Biology and Toxicology, Manipal School of Life Sciences, Manipal Academy of Higher Education, Manipal, 576104 India; 3 Department of Cell and Molecular Biology, Manipal School of Life Sciences, Manipal Academy of Higher Education, Manipal, 576104 India; 4 Department of Pharmacology, Manipal College of Pharmaceutical Science, Manipal Academy of Higher Education, Manipal, 576104 India; 5 Dr. Reddy’s Institute of Life Sciences, University of Hyderabad Campus, Gachibowli, Hyderabad, 500046 India; 6 Shri Dharmasthala Manjunatheshwara (SDM) University, Manjushree Nagar, Sattur, Dharwad, 580009 India

**Keywords:** Anticancer, Glioblastoma, Histone deacetylase, Hydroxamic acid, Isoform selective

## Abstract

Epigenetic modifications play crucial roles in glioblastoma growth and aggressiveness, with key regulators including histone deacetylases (HDACs), histone acetyltransferases (HATs), and methyltransferases. Targeting these epigenetic alterations has emerged as a promising therapeutic strategy, utilizing DNA methyltransferase (DNMT) inhibitors, HDAC inhibitors (HDACis), and miRNA-based therapies. HDACis, whose effect on p53, p21, Bax, and Bcl-2, have gained significant interest because of their ability to restore the expression of tumor suppressor genes, thereby inducing apoptosis and overcoming therapeutic resistance. Our study demonstrated that a novel hydroxamic acid analogue, compound 3B, effectively inhibited glioma cell (C6) proliferation and exhibited potent anticancer activity. Compound 3B induced G2/M phase cell cycle arrest, increased apoptotic cell populations, and significantly reduced colony formating efficiency. Confocal imaging revealed nuclear condensation and elevated reactive oxygen species (ROS) levels, indicating oxidative stress. Western blot analysis confirmed that HDAC inhibition increased AcH3K9 protein levels. Further, studies in in vivo xenograft model and allograft C6 Wistar rat model revealed strong antitumour activity, suggesting that compound 3B is a promising therapeutic candidate for glioblastoma treatment.

**Supplementary Information:**

The online version contains supplementary material available at 10.1186/s40478-025-02194-7.

## Introduction

Glioblastoma (GBM) is one of the most prevalent primary brain tumor. It predominantly affects individuals aged 50–60 years and accounts for approximately 70% of all brain and nervous system cancers [9]. Memory-related problems, headaches, personality changes, seizures, speech issues, and paralysis or numbness in the arms, legs, and face are all symptoms of aggressive primary tumors of glial cells [10]. Current monotherapy options for GBM are limited due to dysregulated growth pathways, poor pharmacokinetics, and toxicity concerns. Extensive clinical trials have led to the approval of the DNA alkylating agent temozolomide (TMZ) as an adjuvant therapy for GBM. However, its limited efficacy has driven researchers to explore novel therapeutic alternatives [2, 32, 33]. One of the key factors contributing to glioma aggressiveness is altered epigenetic regulation. Epigenetic modulation, particularly that involving histone deacetylases (HDACs) and histone acetylases (HATs), plays a crucial role in tumor progression [5].

HDACs play crucial roles in chromatin condensation and transcriptional silencing by altering gene expression *via* elimination of acetyl groups from histone and non-histone proteins [15]. In the human genome, eighteen HDACs are classified into two primary categories: NAD-dependent sirtuins (class III) and zinc-dependent HDACs (classes I, II, and IV) [18]. In GBM, dysregulation of HDACs contributes to the silencing of proapoptotic and tumor suppressor genes. HDAC1 and HDAC2 have been extensively studied for their critical roles in memory and cognition. Although high HDAC1 and HDAC2 expression is related to glioma aggressiveness and proliferation, to date, no drugs have been approved for glioblastoma treatment [11, 28].

Consequently, HDAC inhibition has emerged as a promising strategy to counteract glioma aggressiveness and improve therapeutic outcomes [1]. HDAC inhibitors function by disrupting enzyme activity, thereby reactivating tumor suppressor genes implicated in apoptosis, cell cycle arrest, and the inhibition of angiogenesis and metastasis in various cancers [22, 23, 26]. Mesenchymal GBM cells exhibit resistance to multiple therapies and recurrence. Kotian et al. [17], demonstrated that HDACis can induce apoptosis and disrupt the cell cycle. Their study also showed that combining HDACis with the epithelial transcription factor Grainyhead-like 2 (GRHL2) enhances cellular reprogramming towards apoptosis [17]. Clinically, several FDA-approved HDAC inhibitors, including suberoylanilide hydroxamic acid (SAHA), belinostat, romidepsin, and givinostat, have shown efficacy in a range of cancers and certain genetic neuromuscular disorders. Panobinostat received FDA approval in 2015 for use in combination therapy in relapsed/refractory multiple myeloma; however, this approval was withdrawn in 2022. Additionally, chidamide has been approved for clinical use in China [16, 19]. However, the therapeutic use of pan-HDAC inhibitors is often limited by side effects such as nausea and headaches and thrombocytopenia. Therefore, developing novel, potent, and selective hydroxamate-based HDAC inhibitors with improved efficacy is crucial. In gliomas, targeting HDAC1 and HDAC2 has been shown to potentially inhibit tumor progression and enhance glioblastoma cell sensitivity to treatment [6].

This study focused on the biological evaluation of hydroxamate based analogues with potent anticancer activity and HDAC1 and HDAC2 inhibitory properties. Through in vitro, C6 xenograft model, and C6 allograft models, we aimed to demonstrate its potential as an effective therapeutic agent for GBM.

## Materials and methods

SAHA and CI-994 were used as positive controls and were purchased from Sigma Aldrich (USA). Propidium iodide stain was obtained from SRL Chemicals (India). The annexin V-FITC apoptosis staining/detection kit was procured from Abcam. Actin phalloidin stain was purchased from Sigma Aldrich (USA). Dulbecco’s modified Eagle’s medium (DMEM) was acquired from Himedia (INDIA).

### Chemical structure

**Fig. 1:**
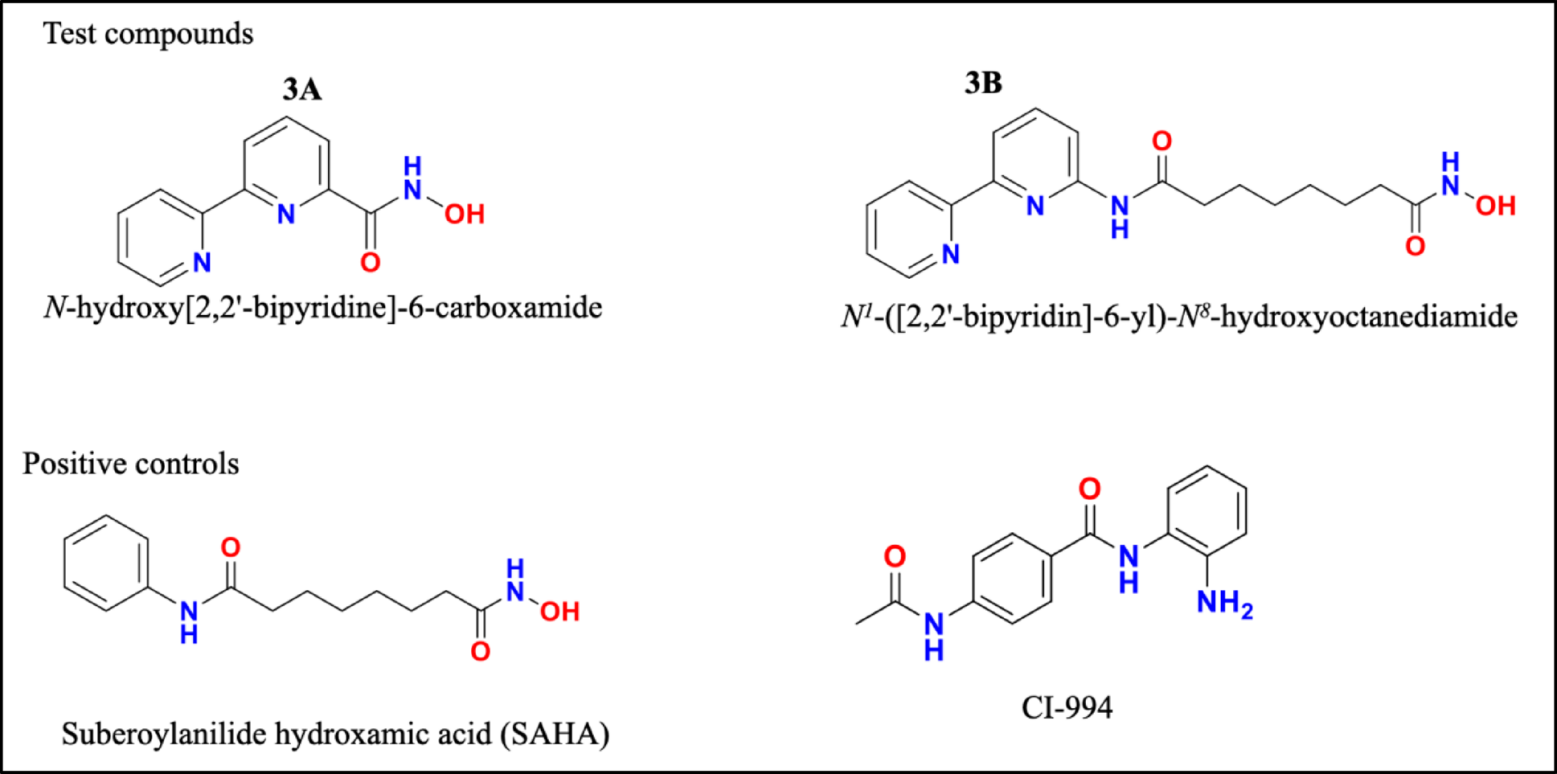
Chemical structures of the test compounds (3A and 3B) and positive controls (SAHA and CI-994)

Two novel hydroxamic acid derivatives (3A-*N*-hydroxy[2,2’-bipyridine]-6-carboxamide and 3B-*N*^′^-([2,2’-bipyridin]-6-yl)-*N*^8^-hydroxyoctanediamide), which are analogues of SAHA, have been designed, synthesized, and characterized previously in our laboratory. Preliminary data support that these compounds are potent HDAC inhibitors, and a docking study revealed that these compounds are potent HDAC1 and HDAC2 inhibitors (Supplementary File 2). The structures of the compounds are given below along with those of positive controls (SAHA and CI-994) (Fig. 1).

### Biological evaluation

Rat-derived glioma cell line (C6) was obtained from the National Centre for Cell Science (NCCS), Pune, India, and cultured in low-glucose DMEM supplemented with 10% FBS. The cell lines were incubated at 37 °C with CO_2_ and the culture medium was replaced every 72 h to support optimal growth.

### MTT assay

Cells were seeded in a 96-well plate and incubated for 24 h. They were then treated with varying concentrations of drugs for 48 h. Following treatment, 20 µL of MTT (5 mg/ml) was added to each well, and the plate was incubated for 4 h. After incubation, DMSO was added to solubilize the formazan crystals. Absorbance was measured at 570 nm and 630 nm using a TECAN multi-plate reader (Austria).

### Cell cycle analysis

The cells were grown in 6 cm culture plates at a density of 1 × 10^5^ cells per 1 mL of medium and incubated for 24 h. The cells were subsequently treated with various concentrations of the test compounds and incubated for 48 h. Following incubation, the cells were collected *via* centrifugation, and the resulting pellet was fixed in 70% ethanol. The fixed cells were then treated with RNase. Propidium iodide was added for staining, and cell cycle analysis was performed using a Partec CyFlow Space flow cytometer (Germany).

### Apoptosis assay

The cells were grown in 6 cm culture plates at a density of 1 × 10^5^ cells/ mL of medium and then incubated for 24 h. Following treatment with various doses of the test compounds, the cells were cultured for an additional 48 h. After treatment, the cells were collected, binding buffer was added, and the cells were stained with annexin V and propidium iodide. The cells were incubated for 10–15 min in the dark, and apoptosis analysis was performed using a Partec CyFlow space flow cytometer (Germany).

### Reactive oxygen species (ROS) measurement

6 cm culture plates were seeded with cells at a density of 1 × 10^5^ cells/ mL of medium. The cells were treated with different drug concentrations for 24 h and then incubated for 48 h. Following incubation, the cells were trypsinized, centrifuged, and rinsed with phosphate buffer saline (PBS). The cells were stained with DCF-DA, incubated for 30 min at 37 °C in the dark. After incubation, the cells were centrifuged, the staining solution was discarded, and the pellet was resuspended in PBS. Fluorescence was analysed using a Partec CyFlow space flow cytometer (Germany).

### Confocal imaging

C6 cells were grown on coverslips in a 6-well plate at a density of 2 × 10^5^ cells/ mL of medium. The cells were maintained until they reached 80–85% confluency, after which they were treated with various doses of the drug and left for 24 h. The media was then removed, and the cells were washed with PBS. The cells were fixed with 4% paraformaldehyde, followed by another wash with PBS. The cells were treated with 0.5% Triton X and 5% BSA for 45 min. The cells were then stained with actin‒phalloidin solution for 1 h, washed with PBS, and subsequently stained with DAPI (10 min), followed by washing with PBS. Coverslips were mounted on slides with mounting medium, after which imaging was performed *via* a Leica SP8 confocal microscope equipped (Germany).

### Colony formation assay

C6 cells were seeded at a density of 100 cells per well and allowed to adhere for 24 h. The cells were then treated with various concentrations of the compound and incubated for 48 h. Following treatment, the medium was replaced every three days until day 14. On the 14th day, the colonies were stained with 0.4% crystal violet for 30 min. The stained colonies were then counted and analysed.

### Western blotting analysis

After being seeded in 6-well plates, C6 cells (2 × 10^5^ cells/well) were exposed to varying doses of the test compounds for 24 h. Following treatment, RIPA buffer was used to lyse the cells. The total protein content was then determined by collecting and centrifuging the lysates. The Bradford assay was used to quantify the proteins. Proteins in equal quantities were separated via SDS‒PAGE and then transferred to a nitrocellulose membrane. Primary antibodies against acetyl-histone H3 (Lys9), Bax and β-actin (all diluted 1:3000) were added to the membranes after they were blocked with 5% BSA. After that, the sections were incubated with secondary anti-rabbit antibodies at a dilution of 1:5000. To visualize the protein bands, the iBrightTM CL1500 Imaging System (USA) was utilized.

### *In vivo* tumor xenograft model

All animal experiments were approved by the Institutional Animal Ethics Committee, Manipal Academy of Higher Education, Manipal (IAEC No. IAEC/KMC/82/2023). The study was carried out at the Animal House Facility under controlled conditions. Male BALB/c nude mice were procured from ACTREC, Mumbai. Male BALB/c nude mice weighing 28–30 g and aged 6–8 weeks were chosen for the study. C6 cells (3 × 10^6^) were injected subcutaneously into each mouse flank to create a xenograft tumor model. Furthermore, the animals were observed once the tumors had grown to a size of approximately 100–300 mm³ and were grouped into the sham control, C6 control and compound 3B (n = 3). The compounds were diluted in a solution of 10% DMSO and 10% Cremophor in Milli-Q water. Compound 3B was administered orally to the treatment group once daily for 7 days at a dosage of 50 mg/kg, whereas the control group received only the solvent used for dissolution. The mice were sacrificed after the treatment period, the tumors were excised, and the weights were recorded. The collected tumors were subjected to histopathological analysis.

### Histopathology

The tissues were first fixed in 4% paraformaldehyde, followed by dehydration and embedded in paraffin. Thin sections, each 4 μm thick, were prepared via a microtome in the vertical plane. These sections were first deparaffinized and then stained for five minutes with Mayer’s hematoxylin and then for two minutes with eosin-phenoxine. The stained sections were subsequently dehydrated and mounted with a neutral resin. Histopathological examination was conducted to assess tumor morphology, including necrosis, tumor cell infiltration, muscle invasion and nuclear morphology. Microscopic analysis was performed with an LX-500 LED trinocular research microscope (Labomed), and images were taken with a MiaCam CMOS AR 6 pro microscope camera.

### In vivo tumor allograft model

This study utilized 12-week-old healthy male Wistar rats weighing between 200 and 250 g to evaluate the effects of compound 3B under both normal and GBM conditions. The experimental protocol was approved by the Institutional Animal Ethics Committee (IAEC No. IAEC/KMC/76/2024) and conducted in compliance with ethical guidelines. The animals were housed under controlled conditions, maintained at a temperature of 23 °C and 50% humidity, and provided *ad libitum* access to food and water. A 12-hour light/dark cycle was followed, and the rats were fasted for one hour prior to the experiment. The animals were anaesthetized and C6 glioma cells (1,00,000/10 µl) were injected into the cerebrum at specific coordinates (AP = 0.36 mm, ML = 3.6 mm, DV = 5 mm). The skull opening was sealed with sterile dental cement, and the skin incision was sutured. Betadine was applied post-surgery to prevent infection and promote wound healing.

The animals were allowed a recovery period of five days to heal and facilitate cell proliferation and tumor growth. The samples were then divided into three groups: sham control, control (C6- diseased control), and test (compound 3B). Test compound solutions were freshly prepared on the day of administration. The test group received an oral dose of compound 3B at 50 mg/kg once daily for seven days. On the 13th day, after the treatment period ended, the Open Field Test (OFT) was performed. Following the completion of the experiments, the animals were euthanized, and their brain tissues were dissected, collected for further analysis.

### Open field test (OFT)

The test area was held in a square arena of 48 cm with high barriers to prevent escape. The arena floor was divided into equal squares, with the centre and periphery zones designated. The rats were placed separately in the centre of the arena and allowed to roam freely for 5 min under low light. Their motions were captured by a video-tracking system. Key behavioural data, time spent in the centre were examined. Between trials, the equipment was washed with 70% ethanol to remove any smell cues. All procedures adhered to ethical rules for animal research.

### Histopathology

Hematoxylin and eosin (H and E) staining was performed to evaluate the tissue toxicity and the procedure followed is outlined in Sect. 2.10.

## Results and discussion

### Cytotoxicity analysis of compounds

**Fig. 2:**
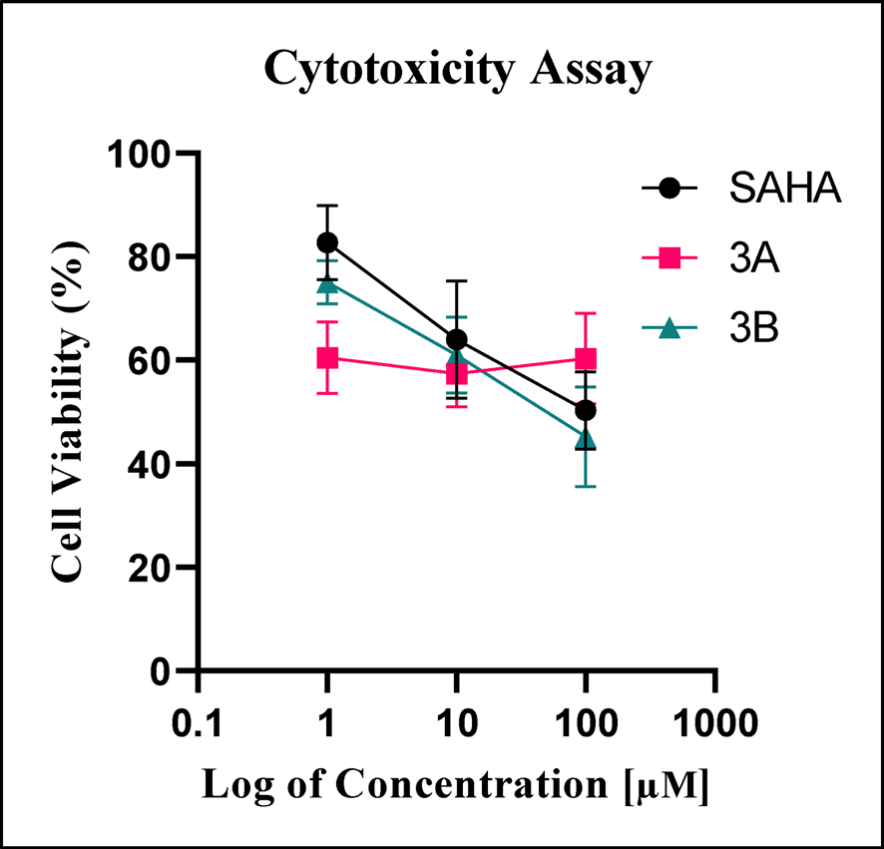
The cytotoxicity of compounds 3A, 3B, and SAHA in C6 glioma for 48 h

The cytotoxicity of the test compounds was evaluated using the MTT assay. SAHA, along with compounds 3A and 3B, exhibited approximately 60% cell viability at a concentration of 10 µM. Therefore, for subsequent experiments, a uniform working concentration of 10 µM was selected (Fig. 2).

### Compound 3B induces G2/M phase arrest

**Fig. 3:**
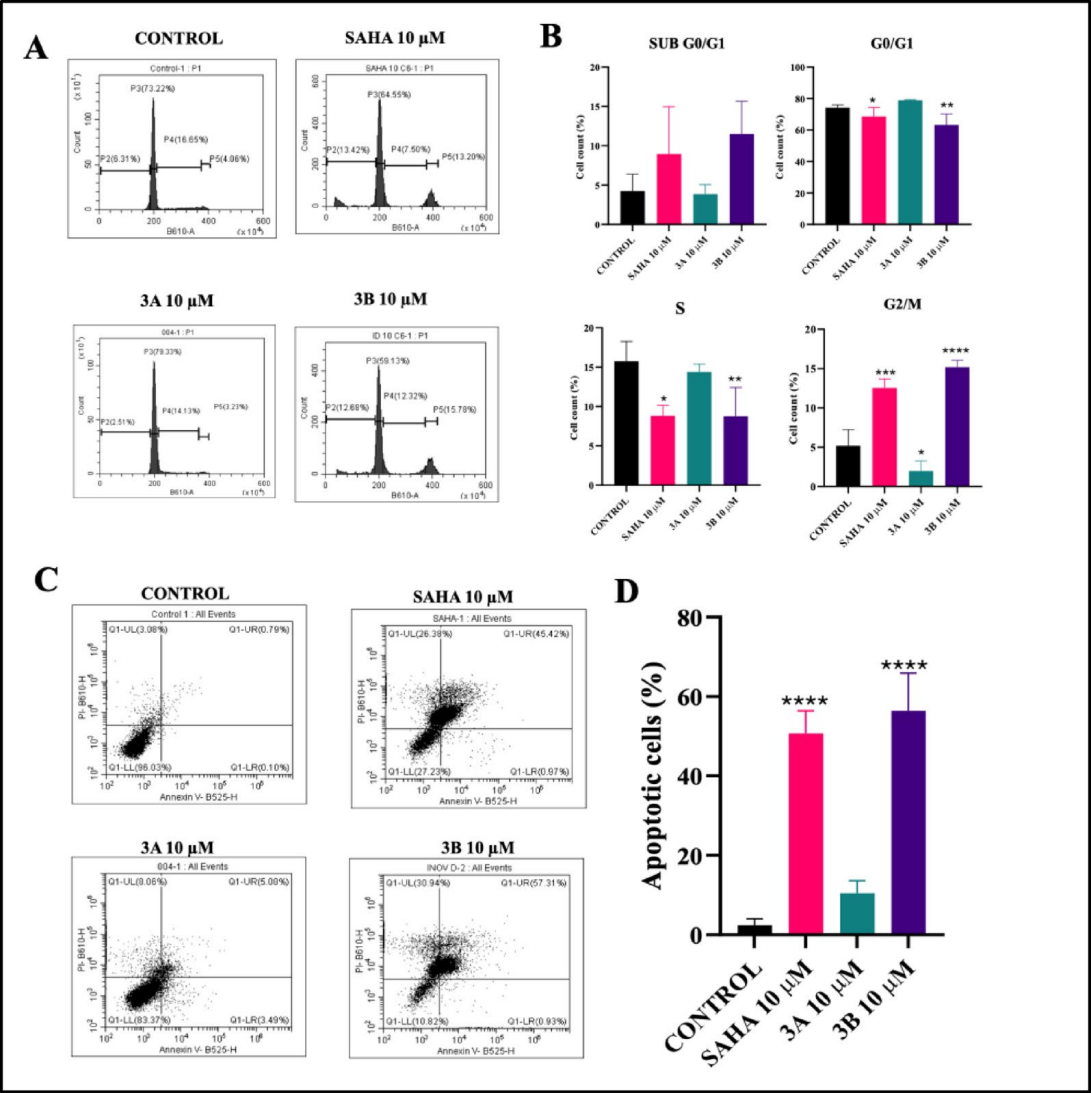
**A** Cell cycle distribution representing flow cytometric histograms of C6 cells at 10 µM concentrations of SAHA, 3A and 3B for 48 h (P2: SUB G0/G1, P3: G0/G1, P4: S and P5: G2/M). **B** Cell cycle analysis of C6 cells at 10 µM concentrations of SAHA, 3A and 3B for 48 h. **C** Representative plots showing apoptotic cell distribution of C6 cells at 10 µM concentrations of SAHA, 3A and 3B. **D** Quantitative analysis of the treated groups compared to the control total apoptotic phases. Data are shown as the mean ± SD (*n* = 2); ****p < 0.0001 (Vs. the control) determined with one-way ANOVA Dunnett’s multiple comparison test. *p < 0.05, **p < 0.01, ***p < 0.001, ****p < 0.0001, (Vs. the control) determined with one-way ANOVA Dunnett’s multiple comparison test

The assay was performed to evaluate the effects of the test compounds on cell cycle progression. Cells were treated with the respective compounds, and the distribution of cells across different phases of the cell cycle (G0/G1, S, and G2/M) was analyzed using flow cytometry. The percentage of cells in each phase was determined to assess the extent of cell cycle arrest induced by the test compounds. The cells were treated with 10 µM of SAHA, compound 3A and 3B for 48 h. Compared with the untreated group, 3B significantly increased the number of cells in the G2/M phase (****p < 0.0001), with 15.57% of the cells remaining in this phase (Fig. 3A, B). These findings suggest that, notably, 3B potently inhibited glioblastoma cell growth and proliferation. The G2/M phase assay indicated that arrest at this stage typically reflects DNA damage or incomplete DNA replication. In a similar study, treatment with Trichostatin A (TSA) markedly reduced cell viability across all three neuroblastoma cell lines. This decrease in viability was closely linked to cell cycle arrest at the G2/M checkpoint.

### Compound 3B induces apoptosis

This assay was performed to check the cells are live, early and late apoptosis and necrotic assay. Annexin V binds to early apoptosis cells and propidium idodie stain binds to the late apototic cells and necrotic cells. Treatment with compounds SAHA, 3A and 3B at a 10 µM concentration induced apoptosis in C6 glioma cells. Notably, compound 3B significantly (****p < 0.0001) increased the percentage of apoptotic cells to 45.68% (Fig. 3C, D). Additionally, the 3B compound demonstrated an apoptotic effect similar to that of SAHA. These findings suggest that compound 3B possesses potent anticancer properties, with the potential to effectively inhibit glioblastoma cell growth. Treatment group cells increased the apoptotic cells after treatment which reflects the increase in apoptotic pathways intrinsic pathway or extrinsic pathway. Therefore, a higher percentage of apoptotic cells in the treatment group indicates the efficacy of the compound in decreasing the cancer cells.

### **Anti-proliferative impact of compounds 3A and 3B**

**Fig. 4:**
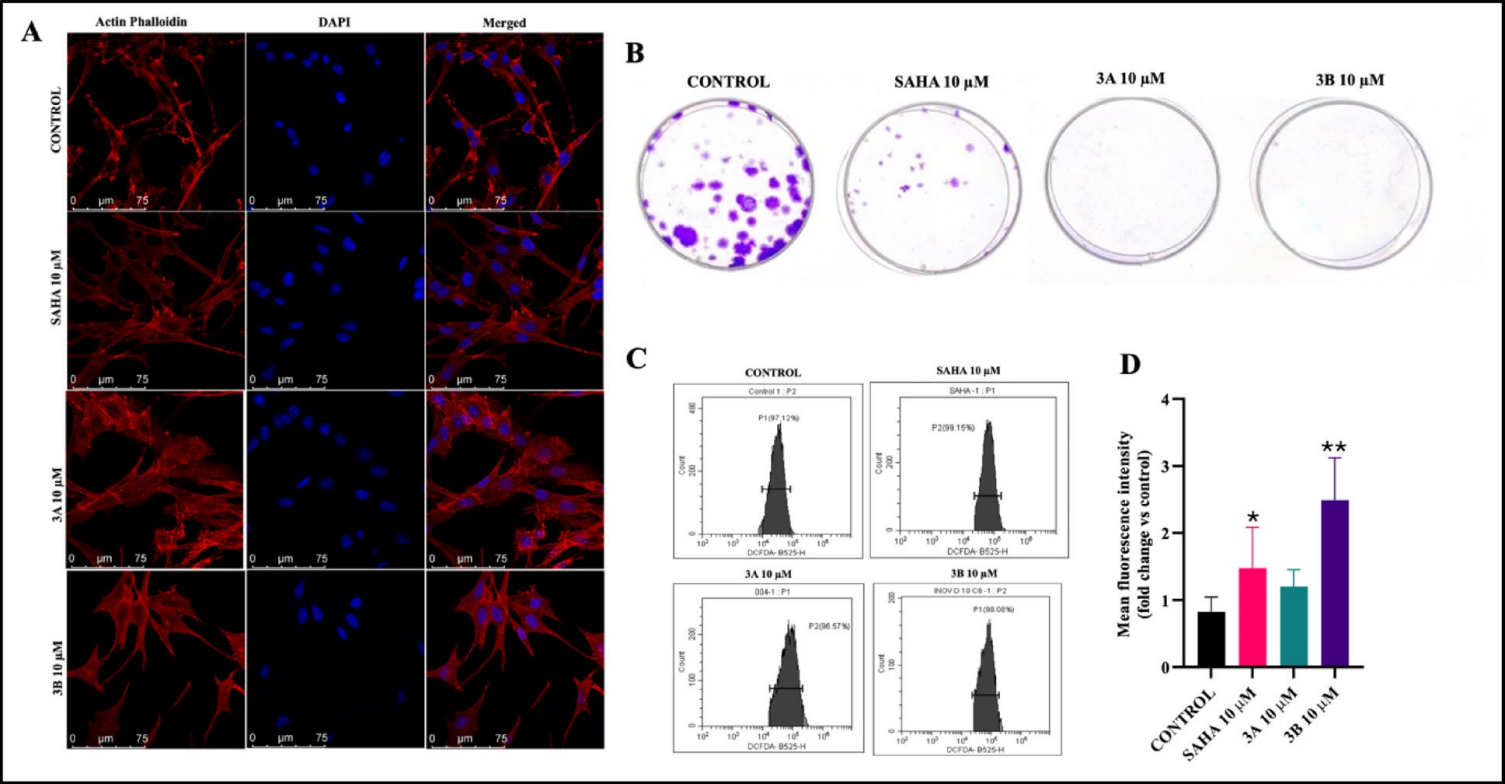
**A** The nuclear and cytoskeleton-stained images by confocal imaging of the C6 cells at 10 µM concentrations of SAHA and 3A, 3B for 48 h (scale bars: 75 μm) **B** C6 glioma cells treated with 10 µM concentrations of SAHA for 48 h for colony formation **C** Representative histogram showing ROS measurement of C6 cells at 10 µM concentrations of SAHA and 3A, 3B for 48 h **D** Quantitively analysis of C6 cells at 10 µM concentrations of SAHA and 3A, 3B for 48 h. Data are shown as the mean ± SD (n = 2); *p < 0.05, **p < 0.01, ns: non- significant (vs. the control). Determined with one-way ANOVA Dunnett’s multiple comparison test

Colony formation assay was performed to identify the cells ability to survive and form colonies. Assay helps to understand drug ability to prevent the cancer recurrence. C6 glioma cells treated with SAHA, compounds 3A, and 3B at a concentration of 10 µM for 48 h showed a reduction in clonogenic efficiency. Among these, compounds 3A and 3B demonstrated the most potent inhibitory effects, completely suppressing colony formation efficiency and outperforming SAHA (Fig. 4B). Test compounds showed the potent antiproliferative effect against the cells.

### Nuclear bulging of glioma cells

Confocal imaging was carried out using DAPI and phalloidin to perform the cellular structure and morphology. The phenotypic characteristics of the cells, including mechanical stability and bulging, were analysed following treatment with the test compounds at 10 µM for 24 h. This ability was assessed via the use of actin-phalloidin (F-actin fluorescent probe) and DAPI (nuclear stain). These stains allowed the evaluation of whether the cytoskeleton and nuclei were affected in C6 cells treated with the compounds. The results indicate that, compared with the control, C6 cells treated with 3A and 3B were unable to maintain their actin filament structures, leading to cytoplasmic bulging, as the cells lost mechanical stability. These findings suggest that 3A and 3B induced apoptosis could be associated with the disruption of actin filaments (Fig. 4A). The test compounds induced distinct morphological alterations, including cytoskeletal disorganization and nuclear bulging, which are characteristic features associated with apoptosis.

### Compound 3B induces ROS in glioma cells

Cells experience oxidative stress as a response to drug treatment such as cellular stress and apoptosis, triggering various cellular mechanisms to cope with increased reactive oxygen species. Treatment of C6 glioma cells with compounds SAHA, 3A and 3B at a concentration of 10 µM for 48 h led to an assessment of intracellular ROS levels via the fluorescent probe DCF-DA in flowcytometry. The results revealed a significant increase in ROS levels induced by compound SAHA and 3B. These findings may suggest that an increase in the number of apoptotic cells is may be due to ROS generation (Fig. 4C, D).

### Histone acetylation, cell cycle regulation and apoptotic markers assessed by western blotting

**Fig. 5:**
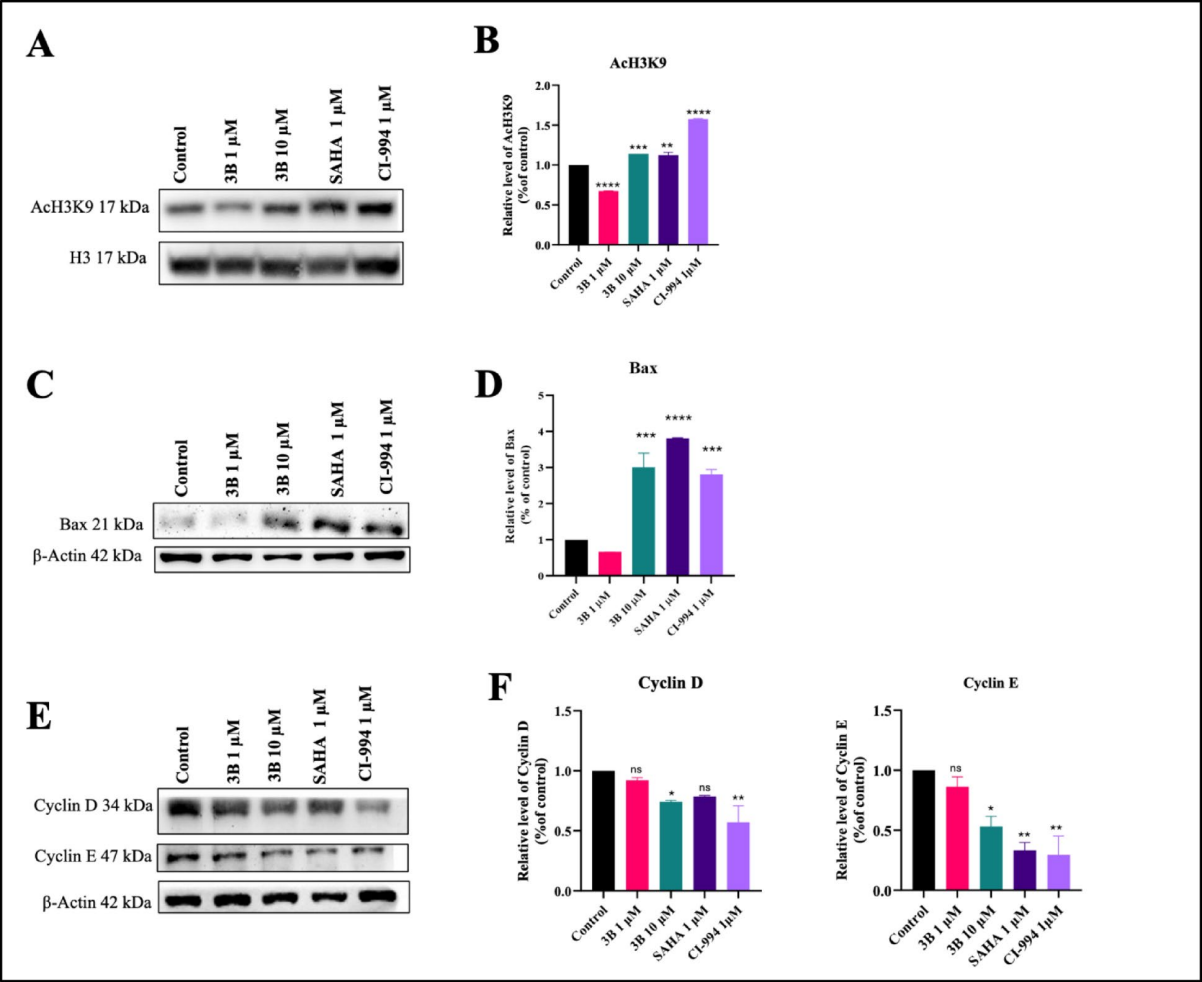
**A** Western blot analysis of compounds on AcH3K9 along with positive control. **B** Densitometric quantification of AcH3K9 in C6 treated with 3B, SAHA, and CI-994 for 24 h compared to the control; **C** Western blot analysis of compounds on Bax along with positive control. **D** Densitometric quantification of Bax in C6 treated with 3B, SAHA, and CI-994 for 24 h compared to the control; **E** Western blot analysis of compounds on Cyclin D and E along with positive control. **F** Densitometric quantification of Cyclin D and E in C6 treated with 3B, SAHA, and CI-994 for 24 h compared to the control; Data are shown as the mean ± SD (n = 2); ***p < 0.001, ****p < 0.0001 (vs. the control) determined with one-way ANOVA, Dunnett’s multiple comparison test

Western blotting analysis is performed to assess changes in protein expression in response to treatment. This technique helps elucidate the underlying molecular mechanisms and signaling pathways involved. We have previously reported that compound 3B is a potent HDAC1 and HDAC2 inhibitor [24]. Hence, in this study, we focused on the effect of compound 3B on acetylation in C6 glioma cells. Western blot analysis was performed to evaluate the dose-dependent hyperacetylation of histone H3K9 proteins over a 24-hour period. These findings revealed a progressive increase in histone H3K9 hyperacetylation with increasing drug concentration, in contrast with the control H3 total. Results indicating increased acetylation of H3K9 without any change in total H3 protein levels (Fig. 5A, B). These results revealed the potential of compound 3B as HDACi. Additionally, apoptosis induction was assessed by analysing the expression of the proapoptotic protein Bax. Western blot analysis revealed a increase in Bax expression in compound 3B treatment group, indicating that compound 3B has potential anticancer properties. β-actin served as an internal control, maintaining stable expression levels across various drug concentrations, untreated controls, and positive controls (Fig. 5C, D). BAX expression is upregulated during apoptotic pathways, while acetylated histone levels increase when HDAC inhibitors block the deacetylation of histone proteins, resulting in enhanced histone acetylation.

The study also aimed to assess cell cycle progression by evaluating the expression of cyclin D and cyclin E. Study indicated that both cyclin D and E levels were reduced in the treatment groups, suggesting a halt in cell cycle progression. As shown in Fig. 5E and F, the expression of these markers was significantly lower in the treated groups compared to the control, same effects seen with the positive control. Additionally cell cycle arrest could induce cytostatic effects and decreased cellular proliferation.

### In vivo antitumor potency of compound 3B in xenograft model

#### Tumor suppression upon treatment with compound 3B in C6 xenograft model

**Fig. 6:**
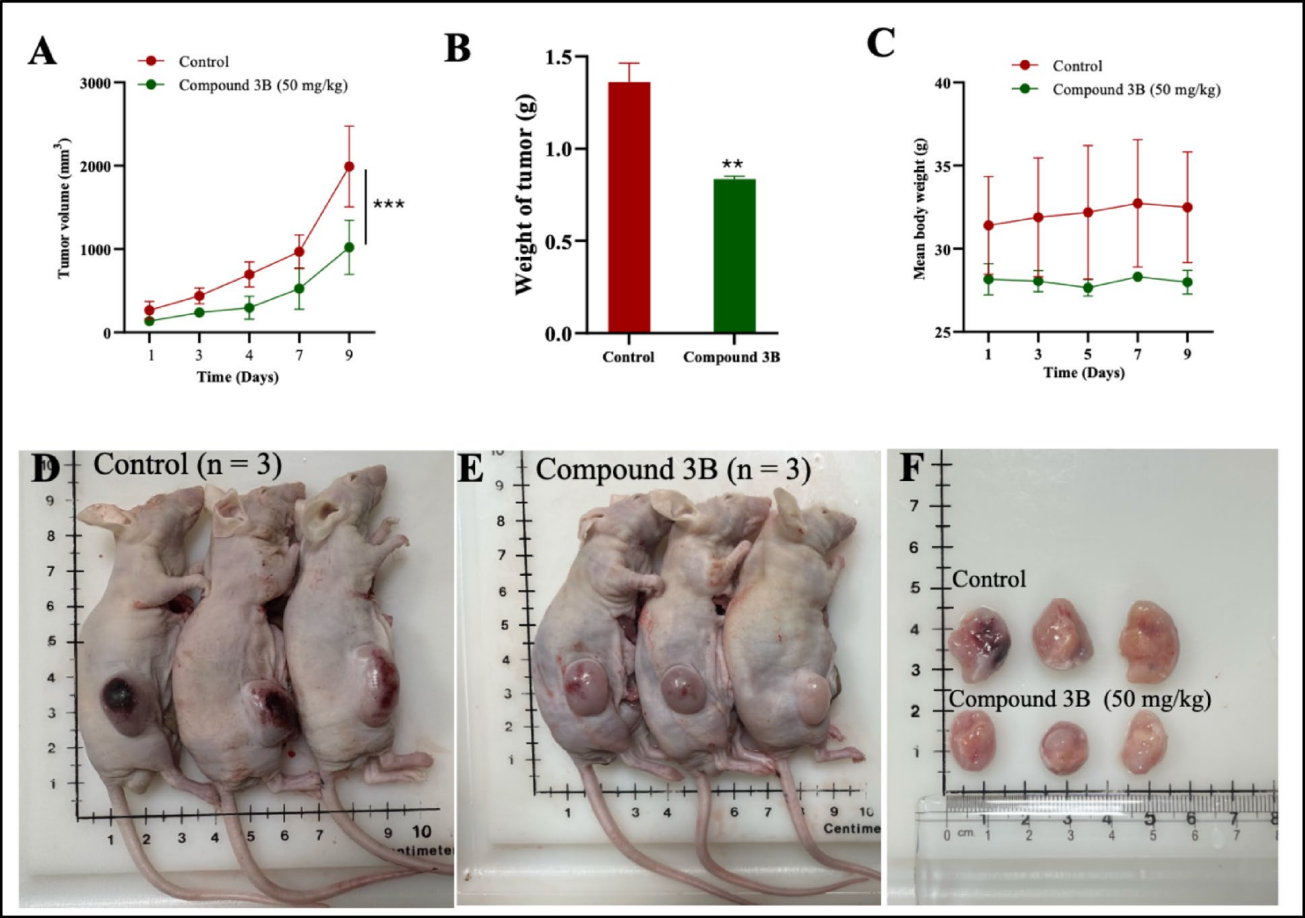
In vivo xenograft antitumor efficacy of compound 3B against C6 xenograft model **A** Tumor volume measurement between control and treated group; **B** Body weight comparison between control and treated group; **C** Tumor weight comparison across control and treated group; **D** Images showing tumor-bearing nude mice in the untreated control group; **E** Images showing tumor-bearing nude mice in the compound 3B (50 mg/kg); **F** Excised tumors from both groups. Data are shown as the mean ± SD (n = 3); ***p < 0.001 (vs. the control) determined with two-way ANOVA for tumor volume and body weight; **p < 0.01, determined with unpaired *t* test

Xenograft model are used to determine the antitumor effects evaluate the in vivo efficacy, of novel anticancer compounds. C6 xenograft model was assessed to determine the efficacy of the drug. Figure 6A and B indicate that 50 mg/kg of compound 3B considerably reduced tumor growth. The treatment group showed no significant body weight loss, indicating that compound 3B was well tolerated in mice at the tested levels (Fig. 6C). Figure 6D and E depict photos of tumor-bearing nude mice from both untreated control and treated groups, while Fig. 6F shows the tumor size comparison between these groups. These results confirm the in vivo antitumour activity of compound 3B. The compound has promising therapeutic potential to inhibit tumor growth or progression.

#### Histopathological validation of the tumor suppression C6 xenograft model

**Fig. 7:**
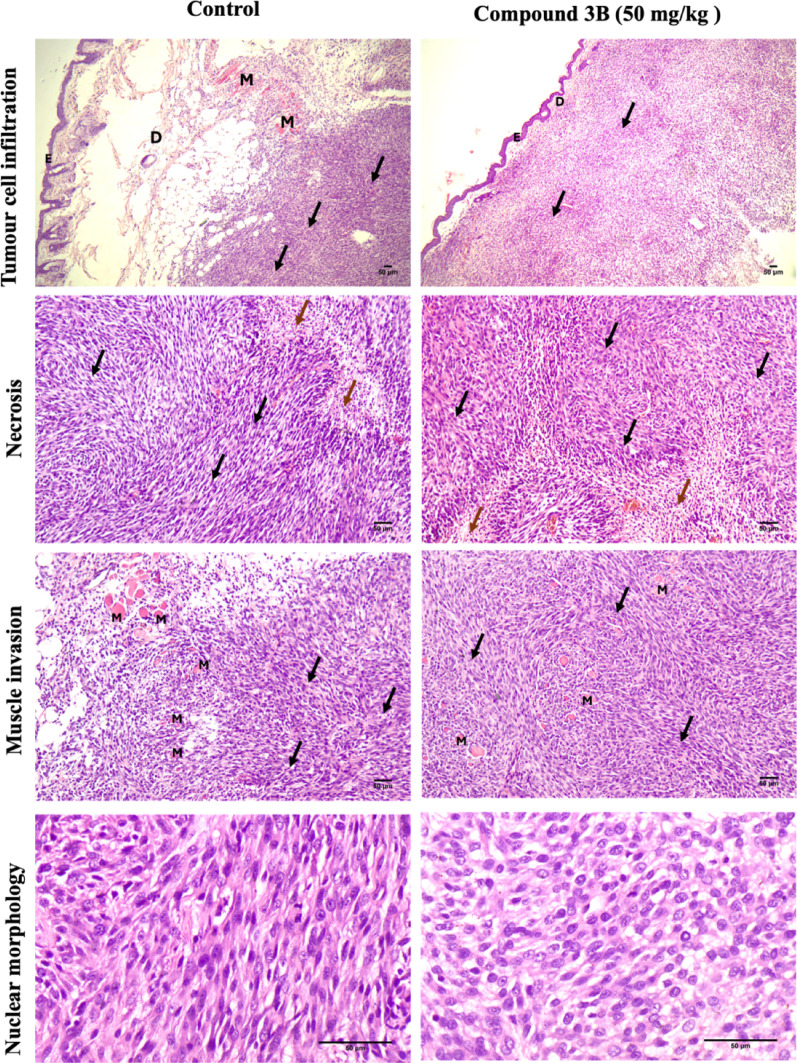
Histopathological assessment after compound 3B treatment in in vivo model. Morphological analysis of tumor control and test compound 3B using H&E staining. Representative images showing H&E-stained sections of the control and treatment group. **A** Tumour cells infiltration into the dermis (40x), **B** Tumour cells and necrosis (100x), **C** Tumour cells with muscle invasion (100x), **D** Mitotic figures, nuclear pleomorphism (400x) (scale bars: 50 μm). Black arrow marks tumor area, brown arrow marks necrosis, Epidermis-E, Dermis-D, Muscle-M

Histopathological analysis is conducted to examine the microscopic structure of tissues, assess tissue integrity, and identify cellular alterations. Histopathological analysis was performed to evaluate tumor-specific characteristics, including tumor cell infiltration, necrosis, muscle invasion and nuclear atypia across the control and test groups. In the control group, tumor cells infiltrated the dermis in clusters, displaying high cellularity and nuclear atypia, such as cellular and nuclear pleomorphism, hyperchromatic nuclei, an increased nuclear‒cytoplasmic ratio, and prominent nucleoli. Tumor cells were also observed to form palisading patterns around necrotic areas, along with evidence of muscle tissue invasion (Fig. 7). Both acute and chronic inflammatory infiltration, as well as mitotic figures, were present in both groups. However, tumor cell infiltration was markedly greater in the control group, whereas the test group exhibited more pronounced necrosis, suggesting differential tumor progression patterns between the two groups. Histopathological assessment after compound 3B treatment in C6 xenograft model scores of each parameter are given in Supplementary File 1. 

### In vivo analysis of tumor growth Inhibition by compound 3B in allograft mice

#### In vivo tumor suppression treatment upon compound 3B in C6 allograft model

**Fig. 8:**
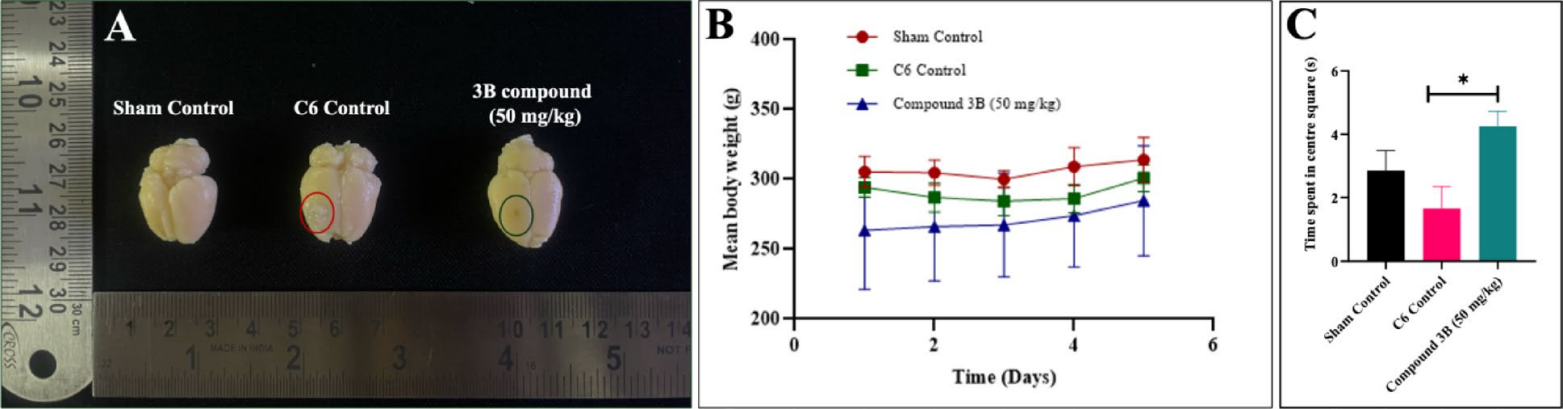
In vivo allograft antitumor efficacy of compound 3B against C6 rat allograft model. **A** Images showing tumor-bearing brain in the sham control, C6 control and test group, **B** Body weight comparison between control and treated group, **C** Comparison between time spent in centre square between sham control, C6 control and compound 3B (50 mg/kg). Data are shown as the mean ± SD (*n* = 3); *p < 0.1 (vs. the control) determined with two-way ANOVA for tumor volume and body weight; **p < 0.01, determined with unpaired t test

The allograft model was employed to evaluate the antitumor efficacy of the test compounds in an immunocompetent and controlled environment. In this study, rats received the treatment, followed by behavioral assessment using the open field test. The animals were sacrificed, and the tumor-bearing brains were carefully excised (Fig. 8A). Throughout the treatment period, the body weight remained stable, as shown in Fig. 8B. No mortality was observed during the study period. The tumor size decreased in the treatment group without affecting body weight, suggesting that compound 3B is highly specific for glioblastoma cells and has minimal toxicity. This study concluded that test compound 3B has effect on glioblastoma in vivo antitumor effect in immunocompetent setting also.

#### Explorative behaviour analysis

The open field test was conducted to assess anxiety-related behaviour in rats by measuring their exploratory activity. The behaviour of the animals in the sham control, C6 control, and treatment group was evaluated. A comparison between the C6 control and treatment groups revealed a significant increase in the time spent in the central square by the treatment group, indicating reduced anxiety levels. This is illustrated in Fig. 8C. The control group exhibited brain tumor growth, whereas the compound-treated group showed a significant reduction in tumor size, indicating the compound’s effectiveness against glioblastoma. No changes in body weight were observed, suggesting minimal toxicity. Additionally, the open field test demonstrated improved behavioral outcomes in the treated group compared to the control.

.

#### Histopathological validation of the tumor suppression C6 allograft model

**Fig. 9:**
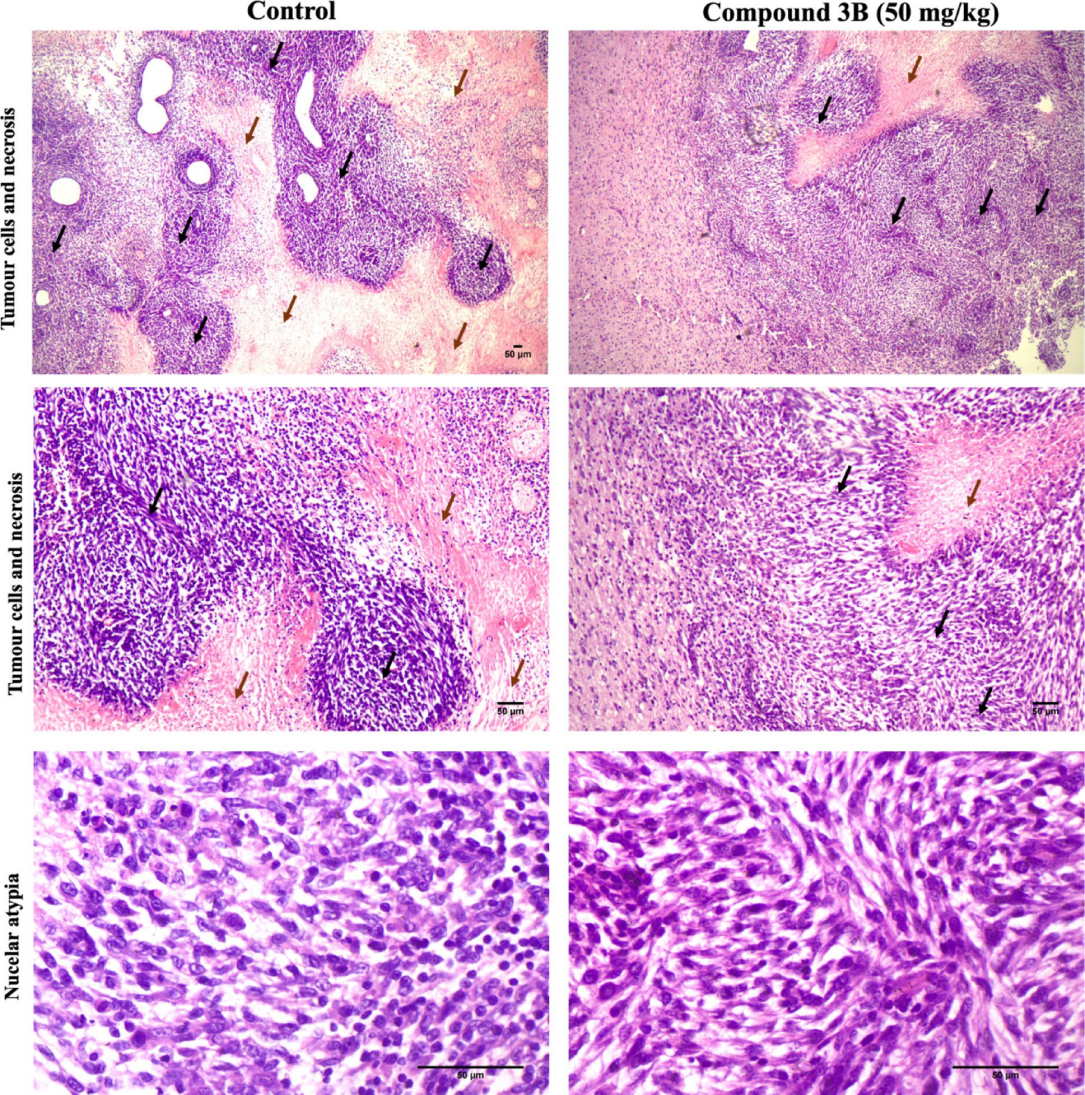
Histopathological assessment after compound 3B treatment in in vivo C6 rat model. Morphological analysis of tumor C6 control and test compound 3B H and E staining. **A** Images of Tumour cells and necrosis with tumor area marked with black arrow and necrosis marked with brown arrow (40x), **B** Images of Tumour cells and necrosis with tumor area marked with black arrow and necrosis marked with brown arrow (100x), **C** Tumour cells with reduced nuclear atypical features (400x) (scale bars: 50 μm)

Histopathology was carried out to check the change in tissue structure and cellular microscopic morphological parameters. Histopathological analysis was performed to examine morphological parameters such as tumor infiltration, necrosis, nuclear atypia, and lymphatic infiltration. In the C6 group, tumor cells infiltrated the cerebrum in clusters and displayed increased cellularity and marked nuclear atypia, including cellular and nuclear pleomorphism, hyperchromatic nuclei, a high nuclear‒cytoplasmic ratio, and prominent nucleoli. In contrast, the test group exhibited only a small area of glial cell proliferation in the cerebrum, with minimal nuclear atypia, such as mild pleomorphism (Fig. 9). Additionally, the test group demonstrated chronic lymphocytic and inflammatory infiltration, potentially indicating that an activated immune response against tumor progression features was absent in the C6 control group. Overall, the test group showed reduced cell proliferation and fewer atypical nuclear features than did the C6 control. Histopathological assessment after compound 3B treatment in C6 xenograft model scores of each parameter are given in Supplementary File 1. 

## Discussion and conclusion

HDACs regulate both histone and nonhistone proteins, playing crucial roles in genome functions such as chromatin assembly, DNA repair, replication and recombination [21, 29]. Additionally, they influence various biological processes, including cell proliferation, differentiation, apoptosis, and senescence. Despite the development of numerous HDACis, their clinical application has been limited due to their high toxicity and low specificity [13]. The broad activity across HDAC isoforms is believed to contribute to toxicity, prompting the development of second-generation with improved selectivity [25].

In this study, the potent anticancer activity of two novel hydroxamic acid analogues against C6 glioblastoma cells. Flow cytometry analysis revealed that compound 3B induced significant G2/M phase arrest, thereby effectively inhibiting cell proliferation. Furthermore, compound 3B significantly increased the number of apoptotic cells comparable to the positive control. Similar study by Buyandelger et al. [4] demonstrated that the HDAC inhibitor MPT0B291 selectively induced cytotoxicity in glioma cells led to G1 phase arrest and apoptosis [4]. Comparable outcomes have also been observed with other HDAC inhibitors such as SAHA and valproic acid, both known to promote cell cycle arrest in glioblastoma cells [7].

In the present study colony formation assays confirmed a notable reduction in clonogenic potential following treatment with compounds 3 A and 3B, underscoring their strong anti-proliferative properties. Confocal microscopy further revealed nuclear bulging, a morphological hallmark of apoptosis, indicating structural alterations in the treated cells.

Compounds 3A and 3B are hydroxamic acid derivatives that feature a zinc-binding group (ZBG), structurally similar to the well-known HDAC inhibitor SAHA. The ZBG, such as hydroxamic acid, is typically conserved among HDACis. However, structural changes in the linker and cap group are critical for achieving isoform selectivity. These compounds usually bind to different residues within the protein, targeting areas such as the catalytic tunnel, surface residues, or binding pocket. Thus, the composition of the cap group and linker, as well as the specific amino acids they interact with, are important factors in determining the selectivity profile of these inhibitors. A linker group, typically hydrophobic, is designed to fit snugly within the catalytic site channel, while the capping group interacts with hydrophobic surface regions and serves as the primary determinant of isoform selectivity [27, 30]. In contrast, pan-inhibitors like SAHA thus exhibit lower isoform selectivity. Importantly, isoform-selective HDAC inhibitors offer pharmacological advantages over pan-inhibitors, including reduced off-target effects and more precise epigenetic modulation. Pan-inhibitors tend to cause widespread epigenetic changes, whereas selective inhibitors allow for targeted epigenetic regulation [3].

In our previous study, we investigated the effects of the same novel compounds on neuroblastoma and found that they inhibited HDAC1 and HDAC2 activity. Western blot analysis revealed increased levels of acetylated H3K9, confirming the inhibition of HDACs [24]. Our current research demonstrates that compound 3B induces a similar pattern of histone H3K9 acetylation in glioblastoma cells, while maintaining unaltered levels of histone H3 in both control and treated groups. Suggesting that compound 3B may serve as potent inhibitor of HDAC1 and HDAC2. Western blotting analysis also showed the upregulation of the pro-apoptotic protein Bax, reinforcing its apoptosis-inducing capabilities. These results align with Wang et al. [31] study, who investigated DMC-HA, a hydroxamic acid-based curcumin derivative designed to enhance HDAC inhibition and to inhibit the anti tumor efficacy on glioblastoma [31]. Likewise, Mikael [20] reported that treatment with mocetinostat, a known HDACi, induce apoptosis through Bax upregulation and downregulation of anti-apoptotic proteins such as Bcl-2 and Bid (Mikeal, 2024).

Study also focused on evaluating the cyclin D and E expression, compounds showed decrease in the expression indicating the cell cycle arrest. Additionally, we analysed the expression of Bax (a pro-apoptotic protein), acetylated histone H3 at lysine 9 (AcH3K9), and total histone H3 levels. We observed an increase in Bax and Ac-H3K9 levels, while total H3 remained unchanged. These findings suggest that compound 3B induces cell cycle arrest, upregulates pro-apoptotic signaling, and promotes histone acetylation without altering total histone levels. Downregulation of HDAC5 inhibits proliferation, induces apoptosis, and causes cell cycle arrest in G1 phase by altering the expression of key regulatory proteins like bcl-2 and cyclin D1/CDKs [8]. TSA, known HDACi induce cell cycle arrest and apoptosis through pathways involving p21 induction and modulation of cyclins D and E, inhibiting proliferation including in lymphatic endothelial cells [14].

Furthermore, in our study, in vivo xenograft experiments revealed that compound 3B significantly reduced tumor size without causing any adverse effect on body weight. Histopathological analysis showed higher tumor cell infiltration in the control group, while the treatment group exhibited increased necrosis, indicating the compound’s efficacy. Additionally, the in vivo antitumor activity of compound 3B was evaluated using a C6 allograft model in Wistar rats. The treatment resulted in a noticeable reduction in tumor volume, and histological examination confirmed greater tumor cell infiltration in the control group compared to the treated group. A similar study by Buyandelger et al. [4] demonstrated that the HDAC inhibitor MPT0B291 effectively suppressed glioma growth in a U-87MG xenograft model. Mice treated orally with MPT0B291 at doses of 10 and 25 mg/kg showed a significant reduction in tumor size and extended survival, confirming its potent antitumor activity. The upregulation of HDAC proteins has been implicated in glioblastoma progression [4]. TMZ is classic drug given is glioblastoma as approved, TMZ is not effct due to glioblastoma drug resistance one the class is over expression of class I HDAC. Where HDAC1/2/3 proteins were over expressed in glioma cells compared normal cells. Such studies shows that HDAC inhibition would help in glioma growth inhibition [12].

In conclusion, compound 3B exhibits strong in vitro and in vivo efficacy in both xenograft and Wistar rat C6 models, indicating its potential as an effective therapeutic agent for glioblastoma. Further, its therapeutic scope may extend to other neurological disorders associated with HDAC overexpression.

## Supplementary Information

Below is the link to the electronic supplementary material.

Supplementary Material 1

Supplementary Material 2

## Data Availability

No datasets were generated or analysed during the current study.
