# Supplementary material for: Targeting glioblastoma with HDAC inhibitors: insights into hydroxamic acid-based therapeutic strategies
Source: Acta Neuropathol Commun. 2025 Dec 2;14:9. doi: 10.1186/s40478-025-02194-7 (PMC12776992; doi:10.1186/s40478-025-02194-7)
Supplement: Supplementary file 1 — Supplementary Material 1 [file 40478_2025_2194_MOESM3_ESM.docx]

**Supplementary File 1**

**Targeting Glioblastoma with HDAC Inhibitors: Insights into Hydroxamic Acid-Based Therapeutic Strategies**

Padmini Pai^1^, Ipshita Das^1^, Yashaswini Reddy^1^, Babu Santhi Venkidesh^2^, Poonam Bhandari^3^, Manjunath Madalageri^4^, Veeresh Sadashivanavar^4^, Karkala Sreedhara Ranganath Pai^4^, Pallavi Rao^5^, Srinivas Oruganti^5^, Manasa Gangadhar Shetty^1^, Kapaettu Satyamoorthy^6^, Babitha Kampa Sundara^1^*

^1^Department of Biophysics, Manipal School of Life Sciences, Manipal Academy of Higher Education, Manipal- 576104, India

^2^Department of Radiation Biology and Toxicology, Manipal School of Life Sciences, Manipal Academy of Higher Education, Manipal- 576104, India

^3^Department of Cell and Molecular Biology, Manipal School of Life Sciences, Manipal Academy of Higher Education, Manipal- 576104, India

^4^Department of Pharmacology, Manipal college of Pharmaceutical Science, Manipal Academy of Higher Education, Manipal- 576104, India

^5^Dr. Reddy’s Institute of Life Sciences, University of Hyderabad Campus, Gachibowli, Hyderabad- 500046, India

^6^Shri Dharmasthala Manjunatheshwara (SDM) University, Manjushree Nagar, Sattur, Dharwad- 580009, India.

*Corresponding author

Dr. Babitha K S

Associate Professor and Head

Department of Biophysics

Manipal School of Life Sciences

Manipal Academy of Higher Education

Manipal- 576104, Karnataka, India

E-mail address: babitha.ks@manipal.edu

ORCID: <https://orcid.org/0000-0002-9385-9353>

**3.8.2 Histopathological validation of the tumor suppression C6 xenograft model**

**Table 1.** Histopathological assessment after compound 3B treatment in C6 xenograft model scores of each parameters

| Sample code | Tumour cell infiltration | Necrosis | Muscle invasion | Nuclear atypia features |
| --- | --- | --- | --- | --- |
| C1 | +++ | - | Muscle tissue not seen | ++ |
| C2 | +++ | ++ | Muscle tissue not seen | ++ |
| C3 | +++ | - | ++ | ++ |
| T1 | +++ | + | Muscle tissue not seen | ++ |
| T2 | ++ | ++ | Muscle tissue not seen | + |
| T3 | ++ | ++ | ++ | ++ |

**- Nil, + mild, ++ moderate, +++ severe**

**3.9.3 Histopathological validation of the tumor suppression C6 allograft model**

**Table 2.** Histopathological assessment after compound 3B treatment in C6 allograft model scores of each parameters

| Sample code | Tumour cell | Necrosis | Nuclear atypia features |
| --- | --- | --- | --- |
| C1 | **++** | **++** | **++** |
| C2 | **++** | **++** | **++** |
| C3 | **++** | **++** | **++** |
| T1 | **+** | **-** | **+** |
| T2 | **+** | **+** | **+** |
| T3 | **+** | **+** | **+** |

**- Nil, + mild, ++ moderate, +++ severe**
