# Supplementary material for: Targeting glioblastoma with HDAC inhibitors: insights into hydroxamic acid-based therapeutic strategies
Source: Acta Neuropathol Commun. 2025 Dec 2;14:9. doi: 10.1186/s40478-025-02194-7 (PMC12776992; doi:10.1186/s40478-025-02194-7)
Supplement: Supplementary file 2 — Supplementary Material 2 [file 40478_2025_2194_MOESM4_ESM.docx]

**Supplementary File 2**

**Title: Targeting Glioblastoma with HDAC Inhibitors: Insights into Hydroxamic Acid-Based Therapeutic Strategies**

1. ***In silico* analysis of test compounds**

**1.1. Methodology Molecular docking**

Protein structures were retrieved from the Protein Data Bank and prepared using the Protein Preparation Wizard. Ligands were prepared using the LigPrep module, and receptor grid boxes were generated using the Glide module. Molecular docking was then performed using Glide. 2D structures of ligands were used for the study. The compounds were docked against the HDAC isoforms 1-11 (classical HDACs) using the Maestro 11.4 (Schrodinger Inc.).

**1.2 Results of molecular docking**

To estimate the binding potential of compounds 3A and 3B with the active sites of classical HDACs (1 to 11 isoforms) the *in silico* docking studies were performed. Compound 3B demonstrated interactions with all isoforms, while showing the strongest binding affinity with HDAC2 (-12.77 kcal/mol). Its binding affinity was comparable to that of the positive control, SAHA, and compound 3A, which had a binding energy of -12.04 kcal/mol and -10.28 kcal/ mol respectively (**Table 1**). Having comparatively better interaction with HDAC2, the 2D interaction data of 3A and 3B with HDAC2 protein is disclosed and both show that their hydroxamate group forms a metal ion interaction with the catalytic Zn^2+^ ion **(Supplementary Fig. S1).**

**Table 1:** XP dock scores (kcal/ mol) of the compounds 3A, 3B and positive control SAHA with all the classical HDAC isoforms.

| **Compound**  **code** | **HDAC1**  **(4BKX)** | **HDAC2**  **(4LY1)** | **HDAC3**  **(4A69)** | **HDAC4**  **(2VJQ)** | **HDAC5**  **(Q9UQL6)** | **HDAC6**  **(3PHD)** | **HDAC7**  **(3ZNR)** | **HDAC8**  **(1T69)** | **HDAC9**  **(Q9UKV0)** | **HDAC10**  **(6UII)** | **HDAC11**  **(Q96DB2)** |
| --- | --- | --- | --- | --- | --- | --- | --- | --- | --- | --- | --- |
| **SAHA** | -4.43 | -12.04 | -1.18 | -8.29 | -1.17 | -3.52 | -8.24 | -9.91 | -2.24 | -8.97 | -7.73 |
| **3A** | -4.08 | -10.28 | -2.24 | -6.50 | -3.13 | -5.57 | -7.70 | -8.59 | -3.95 | -5.66 | -6.12 |
| **3B** | -6.90 | -12.77 | -4.06 | -8.93 | -5.92 | -3.90 | -9.80 | -9.59 | -5.10 | -8.99 | -7.71 |


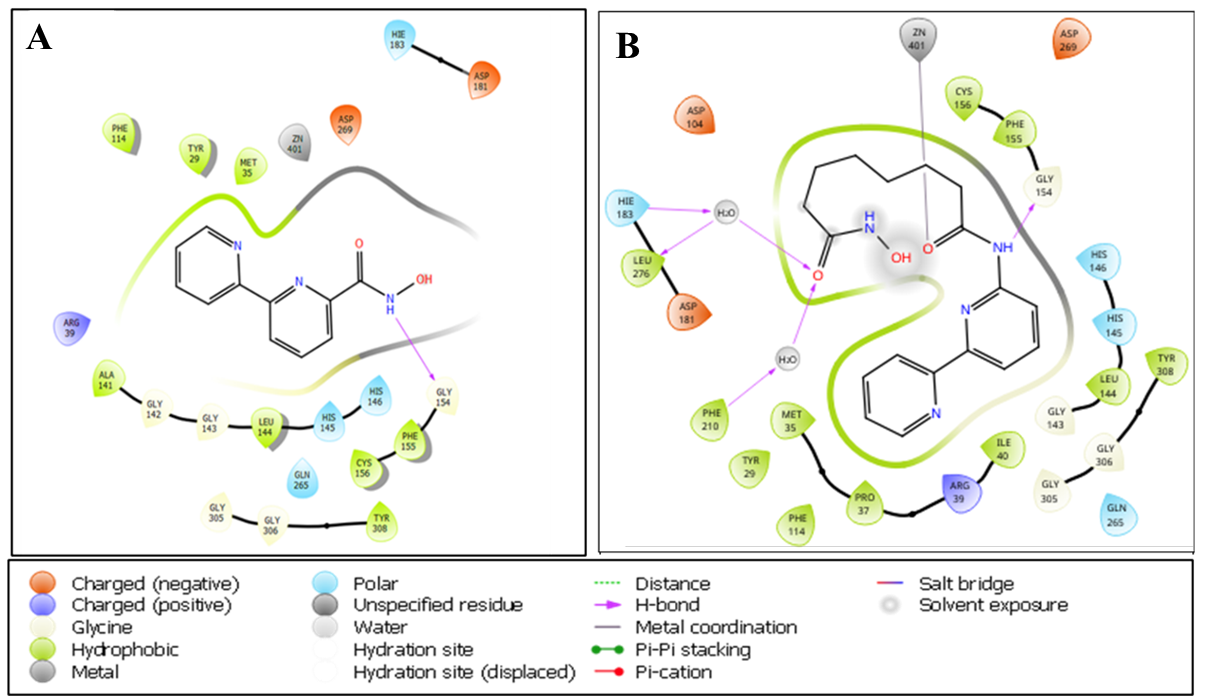


**Supplementary Fig. S1**: Two-dimension interaction diagram of HDAC2-3A and HDAC2-3B complex.

**1.3 Methodology for molecular dynamic simulation**

Molecular Dynamic (MD) simulation was conducted for compounds 3A and 3B in complex with HDAC2 protein to enhance our insights into the stability of their interactions. The Desmond module of Schrodinger was employed for the MD simulations, following a three-step workflow as mentioned by Pai et al., in 2022.

**1.4 Results of molecular dynamic simulation**

Molecular docking studies are limited to predicting ligand interactions at the receptor active site in static conditions, providing docking poses without capturing dynamic atomic movements over time. To address this, MD simulations utilize Newton's equation of motion to estimate atomic motions over time, providing insights into the ligand’s binding state under physiological conditions.

*In silico* molecular dynamics (MD) simulation was performed in addition to the docking study for the HDAC inhibitors 3A and 3B. It was conducted to evaluate the stability of the predicted binding modes. Protein-ligand interaction stability during the simulation was evaluated using RMSD (root mean square deviation) analysis. Following the docking results, Compound 3A, exhibiting the XP dock score (-10.28) toward HDAC2, was subjected to 100 ns MD simulation to predict its affinity for HDAC2. MD trajectory analysis was employed to assess the interaction between protein and ligand interactions, deviation from the root mean square value (RMSD), and root mean square fluctuations (RMSF). RMSD and RMSF provide insights into the equilibrium and fluctuations of the protein complex throughout the simulation. The RMSD plot for 3A-HDAC2 (**Supplementary Figure S2**) revealed slight drifts during specific time intervals (0 to 30 ns) but overall stability from 30 to 100 ns during the simulation was observed. The analysis of the MD simulations for compound 3B indicated almost a stable binding mode in HDAC2 in terms of RMSD. However, major drifts were observed at 0 to 9 ns and 11 to 20 ns as shown in **Supplementary Figure S2**.


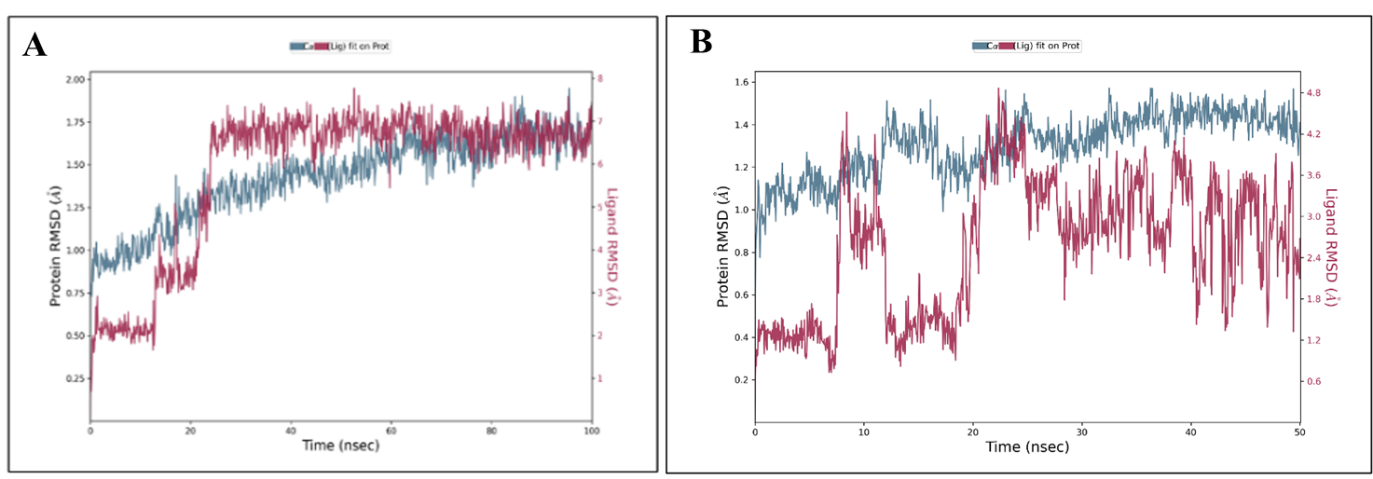


**Supplementary Fig. S2**: Plot presenting the stability of protein-ligand interaction (RMSD) of HDAC2-3A and HDAC2-3B throughout the trajectory.
